# ‘Building back better’ in the context of multi‐hazards in the Caribbean

**DOI:** 10.1111/disa.12545

**Published:** 2022-05-25

**Authors:** Eleanor Jones, Kristinia Doughorty, Pietra Brown

**Affiliations:** ^1^ Independent Researcher, Department of Geography and Geology University of the West Indies Mona, and Environment and Disaster Risk Management Strategist, Environmental Solutions Limited Jamaica; ^2^ PhD Candidate, Department of Geography and Geology University of the West Indies Mona, and Environmental Analyst, Environmental Solutions Limited Jamaica; ^3^ Independent Researcher, Department of Geography and Geology University of the West Indies Mona and Environmental Analyst, Environmental Solutions Limited Jamaica

**Keywords:** build back better, Caribbean, governance, recovery needs, resilience, risk reduction, social inclusion

## Abstract

The multi‐hazard vulnerability of Small Island Developing States in the Caribbean has underpinned the repeated saga of destructive natural and anthropogenic events that have disrupted land, livelihoods, the economy, and society over the past several decades. Preparedness and response have been the focus of national governments and regional entities and the repeated battering calls into question the concept of recovery and ‘building back better’. This paper examines the concept of recovery and ‘building back better’ in the context of the Caribbean, paying particular attention to the experience of the selected countries of Antigua and Barbuda, the Bahamas, Dominica, Guyana, Jamaica, and Saint Vincent and the Grenadines. These nations have recently been impacted by different disasters, ranging from storms to earthquakes to volcanic eruptions. This paper also explores the similarities among the recommendations concerning recovery needs, presenting key insights into suggested approaches for an inclusive people‐centred recovery process that ‘builds back better’.

## Introduction

The multi‐hazardous Latin America and the Caribbean is considered to be the second most disaster‐prone region in the world: some 152 million persons were affected by 1,205 disasters between 2000 and 2019 (OCHA, 2020). The Small Island Developing States (SIDS) of the Caribbean experience hydrometeorological and geological hazards (World Bank, 2012) as they are situated within the tropical cyclone belt and along the border of the Caribbean tectonic plate. The most frequent hazards, including floods, storms, and drought, are expected to be exacerbated by the effects of climate change (Climate Studies Group Mona, 2020), increasing further the vulnerabilities of the region.

Geotechnical hazards also pose a threat: in recent years, earthquakes have had a damaging impact on Caribbean islands. Haiti suffered a 7.0‐magnitude earthquake on 12 January 2010, resulting in at least 316,000 dead or missing people and 80–90 per cent of buildings being damaged or destroyed (DesRoches et al., 2011). More recently, in 2021, Haiti also experienced a 7.2‐magnitude earthquake that claimed the lives of 2,200 persons (UNICEF, 2021a) and Saint Vincent and the Grenadines experienced a volcanic eruption that displaced 12,775 persons (UNICEF, 2021b).

Over the past 20 years, on average, the Caribbean has incurred USD 1.6 billion per year in direct damage due to disasters, resulting in high costs for reconstruction and recovery (World Bank, 2018). Losses owing to disasters in the region include the loss of life, homes, and livelihoods, as well as environmental damage and damage to critical infrastructure and economic activities (Collymore, 2011). The region is also experiencing more frequent extreme events, occurring concurrently or in rapid succession in different states. During the 2017 hurricane season, Hurricanes Irma and Maria (Category 5 events) impacted islands in the eastern and northern Caribbean in the same week of September, producing unprecedented damage (World Bank, 2018). The estimated cost of damage associated with Maria in the subregion was between USD 27 and 48 billion (UNDRR and ECLAC, 2021). While the reconstruction and rehabilitation phase was under way, the region was forced to confront the COVID‐19 pandemic in 2020.

The adoption of the Sendai Framework for Disaster Risk Reduction 2015–2030 by the United Nations General Assembly in 2015 was a part of a global agenda promoting the view that disaster recovery should aim not only to restore what was lost, but also to forge greater resilience across the development sphere (UNDRR, 2015). Priority 4 of the Sendai Framework aims to *build back better* in the spheres of recovery, reconstruction, and rehabilitation (UNDRR, 2015). However, in the face of multiple and concurrent hazards in the Caribbean, recovery requires a holistic approach to improve resilience in the region. Effective recovery planning has therefore become an even more urgent imperative for the journey towards sustainability of the SIDS of the Caribbean, as indeed elsewhere.

## ‘Building back better’ for disaster recovery

Disaster recovery is one of the most complex and least understood concepts in the disaster management cycle (Smith and Wenger, 2007; Rouhanizadeh and Kermanshachi, 2019). Initially, disaster recovery was based on repair and reconstruction activities and the ability of communities to return to normalcy or the pre‐disaster state (Smith and Wenger, 2007; Johnson and Hayashi, 2012). Yet, previous definitions of recovery failed to include social dimensions such as livelihood restoration and well‐being. The United Nations Office for Disaster Risk Reduction defined recovery as:

*The restoring or improving of livelihoods and health, as well as economic, physical, social, cultural and environmental assets, systems and activities, of a disaster‐affected community or society, aligning with the principles of sustainable development and ‘build back better’, to avoid or reduce future disaster risk* (UNDRR, 2017, p. 6).

This definition concentrates on community restoration and community resilience (Rouhanizadeh, Kermanshachi, and Nipa, 2020). As such, disaster recovery relates to resilience‐building. Originally, resilience referred to the ability of persons or groups to return to their pre‐disaster state, recovering with little to no assistance in a short time frame (Manyena et al., 2011). However, *bouncing back* does not translate into or correlate with a change in *the system*, but rather a return to the original position before the hazard event (Manyena et al., 2011). Thus, it may mean a return to a vulnerable state or to a state that caused the disaster initially. Resilience has been modified to consider a ‘bounce forward’ ability, moving, therefore, from a disaster to a more stable, less vulnerable state (Manyena et al., 2011). This advance may be described as a positive transformation of a system or part of a system. Hence, for communities to become more resilient post disaster, recovery efforts should be focused on *building back better* (BBB) to reduce or replace pre‐existing vulnerabilities or underlying conditions of risk (Fernandez and Ahmed, 2019). This requires the inclusion of all affected community members in the recovery process (UNDRR, 2015; Hallegatte, Rentschler, and Walsh, 2018).

## Requirements for ‘building back better‘

Recovery is influenced by the dynamics of power, race, class, gender, past disaster experience, access to resources, and information (Smith and Wenger, 2007; UNDRR, 2015). While recovery activities are carried out following a disaster, effective recovery initiatives require ex ante planning for such events (Rotimi et al., 2009). Experience shows that post‐disaster recovery activities are often ad hoc modifications of previous construction endeavours (Rotimi et al., 2009).

For effective BBB‐centred recovery, legislation is required to ensure that recovery plans and initiatives are implemented. Legislation for BBB can be categorised by compliance and facilitation (Mannakkara and Wilkinson, 2013b). Compliance legislation allows for the enforcement of recovery initiatives that conform to BBB principles such as hazard laws and building codes. It must also be coupled with knowledge and awareness campaigns to ensure that businesses and regulators abide by BBB principles. Facilitation legislation, meanwhile, allows for an efficient recovery process that can account for time‐consuming procedures, access to resources, temporary permit applications, and collaboration with stakeholders (Mannakkara and Wilkinson, 2013b).

Disaster risk financing is also needed to enable successful BBB‐centred recovery, as resilience‐building requires disaster risk reduction (Katongole, 2020). It proactively detects disaster risks and arranges financial resources, policies, and frameworks to build capacity for rapid response and recovery after a disaster (Cubas, Gunasekera, and Humbert, 2020). This increases the resilience of countries to the financial impact of disasters through planning for disaster recovery. The Global Facility for Disaster Reduction and Recovery has recommended budget‐sharing mechanisms for recovery between local and central governments for resilient reconstruction and improved well‐being (Macaskill and Guthrie, 2018). This financial protection reduces liability and permits the provision of emergency support to households through social protection mechanisms, enabling a faster and more transparent disaster response and helping livelihoods to become more resilient (Cubas, Gunasekera, and Humbert, 2020).

The BBB process also requires a more inclusive approach that includes vulnerable groups within society, such as persons with disabilities, special needs populations, low‐income groups, the elderly, and women and children (Cutter, Boruff, and Shirley, 2003; Wisner et al., 2004), which are disproportionately affected by hazards. An inclusive BBB approach includes, therefore, shock responsive and adaptive social protection programmes that respond quickly post disaster (Hallegatte, Rentschler, and Walsh, 2018). Social protection programmes build the resilience of vulnerable groups by increasing their capacity to prepare for, cope with, and adapt to disaster shocks, thereby maintaining their well‐being and reducing the chance of a poverty trap (Bowen et al., 2020). In addition, as vulnerable groups are disproportionately affected, the recovery process must be socially inclusive, mainstreaming gender equality and vulnerable groups in disaster planning (Yumarni et al., 2021).

Post‐disaster recovery primarily focuses on creating a safer, more sustainable built environment. As the inadequate structural capacity of the built environment is a primary reason for damage and loss (Mannakkara and Wilkinson, 2013a), the main aim of BBB is to incorporate risk reduction measures in reconstruction activities, so that pre‐existing vulnerabilities are decreased and resilience to future hazards and climate change is increased (Mannakkara and Wilkinson, 2013a). Furthermore, to allow for the efficient execution of resilient reconstruction activities, there is a need to procure financial and material resources for pre‐disaster initiatives, preventing unnecessary cost surges and the disruption of reconstruction activities (Chang et al., 2011). Social dimensions and reconstruction activities are also linked; restored social linkages can further enhance the reconstruction process (Tierney and Oliver‐Smith, 2012). Communities with strong cohesion and social capital can organise in the absence of government resources and support post‐disaster reconstruction through collective action (Li and Tan, 2019). Community‐led reconstruction activities also provide high levels of satisfaction within communities and promote empowerment among vulnerable groups if facilitated adequately (Crawford and Morrison, 2021). Resilient reconstruction must incorporate, therefore, pre‐disaster recovery planning (PDRP), improved structural quality, social inclusion, and environmental management.

Often overlooked in resilient disaster recovery is environmental management. Like other dimensions of recovery, efforts must be made to restore and improve affected natural environments post disaster. The *greening* of recovery activities to include nature‐based solutions allows for a more resilient society and for communities to ‘bounce forward’ or ‘build back better’ after hazard‐related events (Mabon, 2019). Well‐managed ecosystems provide a buffer to communities, reducing exposure to hazards and increasing their socioeconomic resilience (Sudmeier‐Rieux et al., 2019). The inclusion of nature‐based solutions can also reduce disaster risk and help in adapting to or lessening the impacts of climate change (Farrokhi et al., 2016; Mabon, 2019), resulting in a more resilient community following a disaster.

## Recovery trends in the Caribbean

A regional approach to disaster management in the Caribbean commenced in 1981 with the development of the Pan Caribbean Disaster Prevention and Preparedness Project (PCDPPP) (Bisek, Jones, and Ornstein, 2001; Potter et al., 2015). This intervention followed the impacts of a series of very damaging hurricane and floods across the region, the eruption of the La Soufrière volcano in Saint Vincent and the Grenadines on 13 April 1979, and other dislocating extreme natural hazards. The PCDPPP office was headquartered in Antigua and Barbuda, and the focus was on technical support for preparedness and response and an introductory approach to hazard mitigation and loss reduction initiatives (Kirton, 2013). Several devastating events followed during the 1980s, and hence the Caribbean Community determined that increased collaborative support was required to foster disaster management in member states to stymie the derailing ramifications of disasters for development.

The Caribbean Disaster Emergency Response Agency (CDERA) was established in 1991 to replace the PCDPPP as the regional specialised inter‐governmental body responsible for coordinating emergency response and relief efforts in member states (Bisek, Jones, and Ornstein, 2001). In 2001, a more organised all‐hazards pre‐emptive approach was introduced through the results‐based comprehensive disaster management strategy under the CDERA umbrella. CDERA underwent a name change in 2009, to the Caribbean Disaster Emergency Management Agency (CDEMA), to reflect the increasing focus on all stages of the disaster management cycle—prevention, preparedness, response, and recovery—and the evolving emphasis on vulnerability and risk and loss reduction (Kirton, 2013).

In 1999, CDERA designed a Model National Recovery Framework (MNRF) for its 18 member states. The MNRF was first developed in 1999, but as several new issues emerged relevant to recovery, it was updated in 2014 (Phillips, II, Husein, and Lashley, 2014). While the MNRF has been developed and promoted by CDEMA, recovery planning has not been generally adopted by Caribbean states. Emphasis has traditionally been placed on disaster preparedness and response (Powell, Chakalall, and Hori, 2020). Throughout the region, governments have facilitated the development of ad hoc institutions to manage recovery efforts in an attempt to accelerate rehabilitation and reconstruction, as well as sometimes to reduce bureaucratic delays (Powell, Chakalall, and Hori, 2020). This has resulted in the absence of an institutional and sectoral capacity for PDRP in ministries, agencies, and departments, a slow start‐up with high administrative costs, and duplicated mandates (Powell, Chakalall, and Hori, 2020).

Following Hurricane Maria in 2017, the Government of Dominica developed and enacted the Climate Resilience Act 2018 in response to this devastating event. The Act allowed for the creation of the Climate Resilience Execution Agency for Dominica (CREAD) (Government of the Commonwealth of Dominica, 2020). While CREAD has a four‐year mandate, it is also tasked with transferring its capacities, skills, knowledge, and information. This development signifies/demonstrates a shift within Caribbean states towards a more inclusive and structured recovery process.

## Research design

To assist in conceptualising a framework for Caribbean recovery, a detailed regional assessment of needs and current practices is necessary. A qualitative case study research design was used by this study to understand circumstances in the region, providing an in‐depth analysis of experiences and people or groups (Edmonds and Kennedy, 2017; Creswell and Creswell, 2018; Yin, 2018).

A purposive sampling technique was utilised to select six countries and their respective stakeholders and to garner details of their respective recovery experiences following significant hazards in the past five years. The technique built on previous initiatives to deliberately select potential participants who would offer important information in response to the research questions (Kuzel, 1992; Marshall, 1996; Taherdoost, 2016). Semi‐structured interviews were held to capture information from stakeholders in the selected countries on experiences, challenges, lessons learned, and recommendations. This provided not only a guide for the discussion, but also allowed for the inclusion of additional data captured, essential for the study.

## Case study countries

Six Caribbean nations were selected to conceptualise the recovery context and make recommendations for an improved recovery experience in the region. As the Caribbean is a multi‐hazard region, with differential exposure among territories to hazards, states were chosen according to their geographic location in the Caribbean Basin, the type of hazard, the nature of the territory, and the year of the event. These criteria resulted in the selection of Antigua and Barbuda, the Bahamas, Dominica, Guyana, Jamaica, and Saint Vincent and the Grenadines (see Table 1).

**Table 1:** Criteria used to select the countries for the study

Caribbean countries	Geographic location in the Caribbean Basin	Type of hazard	Nature of the territory	Year of the event
Antigua and Barbuda	East Caribbean	Hurricane Irma	Multi‐island	2017
Bahamas	North Caribbean	Hurricane Dorian	Multi‐island	2019
Dominica	East Caribbean	Hurricane Maria	Island	2017
Guyana	South Caribbean	Flooding	Mainland	2021
Jamaica	Central Caribbean	Tropical Storm Zeta	Island	2020
Saint Vincent and the Grenadines	East Caribbean	Volcanic eruption	Multi‐island	2021

**Source**: authors.

## Data collection and analysis

Data were collected using semi‐structured interviews and a document review. Semi‐structured interviews were conducted with the directors and the deputy directors of disaster management organisations in the six countries. Additional interviews were held with key stakeholders from the ministries of finance, planning, and development programming, and social affairs. The interview questions centred on the participants' disaster experiences, lessons learned, and recommendations for BBB in the region.

A document review served to provide the recovery context in the selected countries as well as to validate information from the interviews. Documents included not only damage and loss reports, but also legal and institutional frameworks and community initiatives in the selected territories.

To facilitate data analysis, interview transcripts were coded manually, using key themes from the literature such as emergency procurement, pre‐disaster financing and recovery planning, resilient reconstruction, and environmental rehabilitation. Codes were also generated for country‐specific post‐disaster experiences. The coded data were subsequently rearranged into main themes and patterns to permit the generation of assertions and recommendations.

## Multi‐hazard impacts of disasters in the Caribbean

As indicated, over the past 10–15 years, the Caribbean has experienced repeated losses and extensive damage due to hydrometeorological hazards, with climate change anticipated to exacerbate the region's risk. Average estimated disaster damage as a ratio of gross domestic product (GDP) is six times higher for countries in the Caribbean, as compared to other states (Ötker and Srinivasan, 2018). The economic cost of disasters in the Caribbean between 1950 and 2016 exceeds USD 22 billion, as compared to USD 58 billion globally (Ötker and Srinivasan, 2018). For some countries, the impact of the disasters surpasses GDP: for example, Hurricane Maria (Category 5, 2017) cost Dominica 225 per cent of its GDP and Hurricane Ivan (Category 4, 2004) cost Grenada 200 per cent of its GDP (Ötker and Srinivasan, 2018).

An increase in the frequency of hazards in the region also underlines the importance of a multi‐hazard approach to BBB. While countries in the region are recovering from one disaster, they are often affected by an additional event, further hindering recovery processes. For instance, Hurricane Dorian (Category 5) made landfall in the Bahamas on 1 September 2019 and remained over the country until 3 September 2019, resulting in estimated damage of USD 2.5 billion and losses of USD 717.3 million. These values were determined by the replacement cost of the assets (IDB and ECLAC, 2020a). Prior to Dorian, Hurricane Matthew (Category 4) had struck the Bahamas in October 2016, causing damage estimated at USD 373 million and losses of USD 145 million (IDB and ECLAC, 2020b). Similarly, in 2021, Saint Vincent and the Grenadines was simultaneously hit by the COVID‐19 pandemic, a severe dengue fever outbreak, volcanic eruptions, and Hurricane Elsa (UNDP, 2021). The nature of these happenings placed a significant strain on the nation's health and economic sectors, hindering the recovery process. To ‘build back better’ in the multi‐hazard context of the Caribbean, therefore, recovery should begin prior to an event and continue throughout the response phase.

## Recommendations for BBB in the Caribbean

Through the document review and consultation process across the region, as articulated above, five main assertions were identified to ‘build back better’:

### Assertion 1: governments need to minimise the need for external resources to meet recovery needs

Most stakeholders highlighted the importance of having local disaster risk financing strategies and resources to support recovery needs. Post disaster, donors may propose ideas and agendas that do not always align with the country's priority activities. As such, they would have to refuse funding when donors were not amenable to modifying the approach. Most funding opportunities are in the areas of climate change adaptation; there are less for measures to facilitate disaster recovery. Proposals sometimes must be repackaged to achieve *buy‐in* and gain attention on an international scale.

Vulnerability to the impacts of non‐climatic hazards needs to be addressed. Not all climate‐induced events may be supported by external sources. Some hazards, such as floods or tropical storms, that result in substantial damage do not attract support from donor agencies, as opposed to major hurricanes that make headlines. This was exemplified by the Guyana floods of 2021, which affected more than 30,000 persons, and by Tropical Storm Zeta in Jamaica in October 2020, which caused an estimated JMD 2 billion of damage but led to limited technical support (such as the development of post‐disaster needs assessments) or financial support from the international community. Furthermore, the process of getting project approval from donors is often time‐consuming and tedious. To circumvent this challenge, government agencies have relied on private sector support to fast‐track recovery efforts. Private sector companies can provide raw materials and technical support with the incentive of monetary tax breaks. Following Hurricane Irma (2017), government agencies in Antigua and Barbuda established a list of contractors and services to utilise in the event of a disaster. Similarly, after Hurricane Maria (2017), Dominica developed a list of pre‐selected contractors and suppliers of services and materials.

To minimise the use of external resources, Caribbean countries can adopt PDRP to accelerate the recovery process and enhance resilience by addressing both short‐and long‐term recovery needs across all sectors and at all levels. This would result in the design of recovery policies, action plans, and programmes and projects that specifically aim to improve resilience for all. To be successful, political support will be required to secure funding for disaster risk financing and multi‐sector engagement. PDRP would also necessitate the development of continuity plans that are essential to keep businesses and agencies functioning at an acceptable level post disaster.

More recently, Caribbean countries impacted by disasters have developed tailored disaster plans. For instance, following Hurricane Maria, the Dominica Climate Resilience and Recovery Plan 2020–2030 was implemented to mobilise the National Resilience Development Strategy. The Plan indicates key areas and tasks to be achieved with an indicative costed plan and potential sources of funding. This is intended to enhance the resilience of Dominica to climate‐related hazards.

### Assertion 2: disaster risk financing

While most Caribbean countries have established a contingency or consolidated fund to be used after a disaster, stakeholders indicated the need for greater investment in risk identification and risk reduction strategies. Of the six countries under review, only Guyana is not a part of the Caribbean Catastrophe Risk Insurance Facility (CCRIF). The CCRIF is a regional parametric insurance policy that can be used in the event of a hurricane, excess rainfall, or an earthquake, providing countries with a cash flow to meet short‐term needs. More recently, in 2021, Jamaica signed up to the World Bank's Catastrophe Bond scheme and Dominica launched a local hurricane parametric insurance facility to augment its resilience to disasters. However, stakeholders indicated the need for a financing strategy that can be used for pre‐disaster risk management. Risk financing strategies can then be used to support continuity plans within government and the private sector. It is recommended that these plans incorporate risk identification and reduction measures for BBB.

### Assertion 3: strengthened social protection services for vulnerable groups

There is a need for a parallel legislative framework and shock responsive system to support households in the long term following a disaster. When disaster offices are activated, immediate support is provided through emergency shelters. Dominica adopted a short‐term mental health and psychosocial support programme after Hurricane Maria. However, affected residents and vulnerable groups need targeted long‐term interventions to increase their resilience to hazards.

Guyana has implemented several ongoing social protection programmes, some of which can be used to help disaster‐affected persons during recovery. One key programme is Public Assistance, which offers support to low‐income families that require temporary, medical, or economic assistance via cash transfers. The National Commission on Disability and the Gender Affairs Bureau have also targeted interventions for vulnerable groups. Similarly, the Jamaica Social Protection Strategy has been established to guarantee the provision of basic income security and essential social services.

Shock responsive social protection services have become increasingly important owing to the growing frequency of extreme dislocating events. Governments are encouraged to consider the unpredictability of the multiple hazards to which the territories of the Caribbean are exposed. The eruption of the La Soufrière volcano on Saint Vincent and the Grenadines during the COVID‐19 pandemic brought to the fore the multi‐layered needs of vulnerable groups, especially as they relate to shelter provision and sustainable social protection services. In response to hazards impacting the country, the Ministry of National Mobilisation, Social Development, The Family, Gender Affairs, Youth, Housing, and Informal Human Settlement (MoNM) implemented the Soufrière Relief Grant scheme to assist vulnerable households affected by the volcanic eruption. The programme provides monthly cash transfers to evacuees staying in shelters, those in private homes, displaced health workers, and other vulnerable populations. The MoNM also partnered with the United Nations Children's Fund to execute mental health and psychosocial support initiatives for schools and communities.

### Assertion 4: stronger coordination is needed among relevant agencies, ministries, and departments

Proper recovery planning requires inter‐agency collaboration among relevant entities and the delegation of tasks and roles before an event. While each Caribbean country has a national disaster office, only a few of them have a specific mandate geared towards disaster recovery. No provision is made for relevant staffing and technical resources. A key recommendation is for clear documentation of roles, responsibilities, and accountabilities with respect to the coordination of recovery activities across government agencies, the private sector, and non‐governmental organisations. Furthermore, with increased clarity on the responsibilities of each institution and the roles of its staff before a disaster occurs, there is an opportunity to reduce duplication and associated costs, and to integrate activities more appropriately.

Following Hurricane Dorian in the Bahamas (2019), the recovery phase was supported by the establishment of a Disaster Reconstruction Authority dedicated to reconstruction, restoration, and recovery post disaster. The Disaster Reconstruction Authority Act, 2019, which governs the operations of this agency, addresses the designation of disaster areas and special economic zones. Although there is some overlap with the Ministry of Public Works, the strengthening of these areas places more focus on disaster recovery and ensures that critical roles are assigned. However, given the long‐term and multidimensional nature of recovery, this approach creates room for progressive neglect of recovery activities, as organisations revert to other tasks over time.

After Hurricane Maria (2017), the Government of Dominica established CREAD to coordinate recovery action following a climate‐related disaster, including the construction, reconstruction, or restoration of physical or other infrastructure and the execution of projects aimed at building national climate resilience. CREAD is also tasked with knowledge transfer and capacity‐building, ensuring the continuity of recovery planning across key ministries and agencies.

### Assertion 5: improved baseline data

A results‐based approach to recovery should be implemented with an emphasis on resilience‐building at the local and national level. All stakeholders lamented the need for sex‐disaggregated baseline socioeconomic data and environmental data to inform vulnerability assessments, disaster risk assessments, land‐use zoning, and social safety nets. Most countries, though, stated that the most recent sex‐disaggregated socioeconomic datasets are post‐census reports. An improved baseline dataset will ensure that communities of all sizes are targeted and that political agendas do not favour those who receive support.

Outwith the periodical census, a Jamaica Survey of Living Conditions (JSLC) is conducted annually to provide the government with information for policy development and planning. The JSLC has evolved since its inception in 1988; currently it collects data on demographic characteristics, household consumption and poverty, health, education, housing, and social protection; the Social Development Commission amasses demographic and asset data at the community level.

A significant portion of the population in the Caribbean lives within the coastal zone, and this proximity to the shoreline increases exposure and vulnerability to climate‐induced hazards (Collymore, 2011). However, limited information is available on key ecosystems such as coral reefs, mangroves/wetlands, coastal forests, and other systems that help to absorb shocks emanating from hydrometeorological hazards. Despite the importance of these ecosystems, they continue to be degraded due to anthropogenic practices. Coupled with the destruction caused by environmental hazards, this has further exposed and intensified the magnitude of damage and loss recorded over time. The stakeholders agreed that there is a need for regulation, enforcement, monitoring, and evaluation of key ecosystem services. Guyana and Saint Vincent and the Grenadines have legislation for the protection and management of key ecosystems (marine and or terrestrial), but there is limited baseline data on the state of the ecosystems and areas requiring rehabilitation or restoration. To ‘build back better’, the protection and rehabilitation of key ecosystems should be considered.

## Conclusion

A considered review of the six case studies in the Caribbean suggests that continual damage and losses due to hazards triggered by extreme natural and human developments are more a function of governance, land use, and human activity, than of the events themselves. In *Disasters by Design*, Dennis Mileti (1999, p. 27) states that: ‘Human beings, not nature, are the cause of disaster losses, which stem from choices about where and how human development will proceed… technology cannot make the world safe from all the forces of nature’.

Processes that create vulnerability are complex and difficult to manage. They include: land, livelihood, and settlement patterns; degradation of natural protective resources; poverty—individual and state, and maybe entailing financial or marginalised conditions; the absence of holistic or integrated planning, which triggers informal/unstructured development, some of which may be encouraged implicitly; and structured developments that focus on short‐term financial/economic interests. As the region continues to plan for multiple hazards, BBB efforts geared towards resilient recovery are encouraged to accommodate PDRP that is inclusive, people‐centred, and environmentally sustainable. PDRP is germane to building resilient nations in the face of multiple hazards.

## Acknowledgements

This paper acknowledges the disaster coordinators in the respective disaster offices that participated in the data collection process and provided background information to supplement the research. We would also like to thank CDEMA for facilitating the interviews with the disaster offices. And we are also grateful to the anonymous peer reviewers for their constructive comments on an earlier version of the manuscript. Finally, we would like to point out that this research received no specific grant from any funding agency in the public, commercial, or not‐for‐profit sectors.

## Data availability statement

The data that support the findings of this study are available from the corresponding author upon reasonable request.
